# Use of Transcriptomics to Identify Candidate Genes for Hematopoietic Differences Between Wujin and Duroc Pigs

**DOI:** 10.3390/ani14233507

**Published:** 2024-12-04

**Authors:** Peng Ji, Ping Wang, Qihua Li, Lin Gao, Yan Xu, Hongbin Pan, Chunyong Zhang, Jintao Li, Jun Yao, Qingcong An

**Affiliations:** 1 Yunnan Provincial Key Laboratory of Animal Nutrition and Feed Science, Faculty of Animal Science and Technology, Yunnan Agricultural University, Kunming 650201, China; 2 Yunnan Tropical and Subtropical Animal Virus Disease Laboratory, Yunnan Animal Science and Veterinary Institute, Kunming 650224, China; 3 Yunnan East Hunter Agriculture and Forestry Development Co., Ltd., Shuifu 657803, China

**Keywords:** Wujin pig, Duroc pig, bone marrow, hematopoiesis, transcriptome analysis

## Abstract

Hematopoiesis is a complex physiological process that ensures renewal of blood cells to maintain normal blood circulation and immune function. Wujin pigs exhibit distinct characteristics such as tender meat, high fat storage, strong resistance to roughage, robust disease resistance, and oxidation resistance. Therefore, using Wujin pigs as models may offer valuable insights for hematopoietic-related studies. In this study, twelve healthy 35-day-old piglets, including six Wujin and six Duroc piglets of similar weight, were selected from each of the Wujin and Duroc pig groups and housed in single cages. After 30 days of feeding, blood and bone marrow samples were collected. Routine blood indices and hematopoietic-related serum biochemical indexes of Wujin and Duroc pigs were determined, and bone marrow gene expression levels were analyzed using transcriptomics. (1) Hemoglobin (Hb) and Mean Corpuscular Hemoglobin Concentration (MCHC) levels in Wujin pigs were significantly higher than in Duroc pigs (*p* < 0.05), and platelet counts and serum Hb levels in Wujin pigs were significantly lower than in Duroc pigs (*p* < 0.05). (2) A total of 312 significantly differentially expressed genes were identified between the pigs. Their functions were mainly related to blood systems, inflammation, and oxidation. Six differentially expressed genes may be related to hematopoietic function. (3) By combining the differential genes screened through sequencing with Weighted Gene Co-expression Network Analysis results, 16 hematopoietic function differential genes were obtained, mainly focusing on immunity, inflammation, and induction of apoptosis functions. Differences were present in the immune and inflammatory responses between Wujin pigs and Duroc pigs, suggesting that differences in hematopoietic function between the two breeds were related to antioxidant capacity and disease resistance.

## Simple Summary

The hematopoietic mechanism is a complex physiological process in organisms, which ensures the continuous production and renewal of blood cells to maintain normal blood circulation and immune function. The Wujin pig is an excellent germplasm resource in China. In this experiment, we investigated the differences in the hematopoietic mechanism between two pig breeds, Wujin pigs and Duroc pigs, and screened the functional genes by transcriptome sequencing technology. Using the Wujin pig as a model can provide new ideas for hematopoietic-related research.

## 1. Introduction

The Wujin pig is a characteristic local pig breed in southwest China. Compared to other domesticated pig breeds, Wujin pigs have the characteristics of tender meat, high fat storage, strong roughage resistance, strong disease resistance, and oxidation resistance [1]. Wujin pigs have been living at high altitudes above 2200 m for a long time, and the low-oxygen environment in the plateau area plays a key role in the development of their resistance to adversity and disease, and antioxidant capacity. Our previous study showed that Wujin pigs possess higher low-oxygen adaptability than Yuedawu pigs [2] and Duroc pigs [3]. At the same time, owing to the high altitude and thin air, high-intensity ultraviolet rays lead to oxidative stress in pigs and lead to the production of reactive oxygen species (ROS), which cause oxidative substances to accumulate in the body or cells, resulting in oxidative damage. Oxidative stress induces changes in hematopoietic capacity [4].

The hematopoietic mechanism is a complex physiological process in the organism that ensures the continuous generation and renewal of blood cells to maintain the normal operation of blood circulation and immune function. As pigs age, the main part of hematopoiesis changes from the liver to the bone marrow. Hematopoietic stem cells (HSCs) exist in bone marrow, which are the source of all blood cells and support balanced hematopoiesis in vivo to meet the needs of physiological blood cell renewal and regeneration under hematopoietic stress [5,6]. Stem cells can develop into a wide range of blood cell precursors, which subsequently differentiate further into a variety of blood cells, such as mature erythrocytes, leukocytes, and platelets [7], and give rise to immune and non-immune organ-resident immune cells [8,9,10]. This differentiation process is regulated by various cytokines and growth factors that are secreted by bone marrow stromal cells, endothelial cells, and other cells into the hematopoietic microenvironment of bone marrow [11,12,13,14,15,16].

Under hypoxic conditions, the kidneys are stimulated to produce erythropoietin (EPO), and the erythroid precursor cells are stimulated to accelerate their maturation and release into the bloodstream, leading to increased numbers of red blood cells and hemoglobin (Hb) content [17]. The hemoglobin content in organisms is affected by many factors, among which altitude is the most important factor, which not only affects the physiological status of the normal organism but also indirectly changes the genetic factors of organisms [18,19]. At the same time, in a low-temperature environment, the human body improves oxygen transportation efficiency by increasing the hemoglobin content and then increasing the metabolic rate to maintain body temperature. People living in high-altitude areas experience some adaptive changes in their hemoglobin molecules, such as changes in the oxygen dissociation curve and suppressed sensitivities to anionic effectors, which make it easier for hemoglobin to combine with oxygen and release oxygen at the tissue level, thus improving the efficiency of oxygen utilization [20]. High altitude can also lead to vasodilation, which can improve the body’s ability to transport oxygen by increasing blood flow [21].

Pigs have a shorter reproductive cycle, larger litter size, a genome similar to that of humans, and an extensive collection of sequencing maps available [22,23]. In addition, the distribution and function of HSCs in pig bone marrow and peripheral blood are similar to those in humans [24]. This makes them suitable for studying human blood diseases, immune system reactions, and blood-related drug testing [25,26,27,28]. The study of Wujin pigs in hematopoiesis is rare. Using Wujin pigs as a model can provide new ideas for hematopoiesis-related research and propose new treatments for a series of blood diseases, such as anemia caused by hypoxia, oxidative stress, and other unfavorable conditions.

## 2. Materials and Methods

### 2.1. Animals and Samples

The experiment involved 12 healthy 35-day-old piglets, including 6 Wujin and 6 Duroc piglets, all of similar weight. The piglets were divided into two groups of Wujin and Duroc according to their breeds. Wujin was labeled as WGB, and Duroc was labeled as DGB. There were six replicates in each group and one pig in each replicate. The experimental pigs were raised at Hunter Agriculture and Forestry Development Co., Ltd. in Yunnan, China, under the same feeding conditions. A basic diet was prepared according to the National Research Council (NRC) Nutrient Requirements of Swine (2012) [29] and the nutritional requirements of pigs (GB/T 39235–2020) [30] (Table 1). The pre-feeding period was 5 days, and the experimental period was 30 days.

### 2.2. Sample Collection

At the end of the experimental period, the piglets underwent a 12 h fasting period. Two tubes (10 mL) of blood were collected from each pig; one tube (with ethylenediaminetetraacetic acid) of blood was used for blood cell analysis, and the other tube (without anticoagulants) was centrifuged at 3000 rpm for 15 min. After standing at room temperature (about 20 °C) for more than 30 min, the serum was collected and stored at −20 °C. After the piglets were slaughtered, the bone marrow of the right front leg and the right rear leg was mixed in a freezing tube, immediately put into liquid nitrogen, and subsequently transferred and stored in a refrigerator (−80 °C).

### 2.3. Determination of Blood Indexes

The blood cell counts were determined using an automated blood cell counter. Serum levels of glutathione peroxidase (GSH-Px) and malondialdehyde (MDA) were determined using commercial kits (Nanjing Jiancheng Bioengineering Institute, Nanjing, China). Serum levels of EPO, Hb, and hypoxia-inducible factor 1 alpha (HIF) were measured using relevant commercial kits (Shanghai Enzyme-Linked Biotechnology Co., Ltd., Shanghai, China).

### 2.4. RNA Extraction, Quantification, and Qualification

RNA was extracted using a SPARK Easy Improved Tissue/Cell RNA Kit (Shandong Sparkjade Biotechnology Co., Ltd., Jinan, China). The RNA concentration and purity were measured using a NanoDrop 2000 (Thermo Fisher Scientific, Wilmington, DE, USA). RNA integrity was assessed using an RNA Nano 6000 Assay Kit on an Agilent Bioanalyzer 2100 system (Agilent Technologies, Santa Clara, CA, USA).

### 2.5. Library Preparation and Sequencing

Sequencing libraries were generated using the NEBNext UltraTM RNA Library Prep Kit for Illumina (Illumina, Omaha, NE, USA), following the manufacturer’s recommendations, and index codes were added to attribute sequences for each sample. The library fragments were purified using the AMPure XP system (Beckman Coulter, Beverly, MA, USA) to select cDNA fragments 240 bp in length. Then, 3 μL USER Enzyme (NEB, Ipswich, MA, USA) was used with size-selected, adaptor-ligated cDNA at 37 °C for 15 min, followed by 5 min at 95 °C before polymerase chain reaction (PCR). PCR was performed using the Phusion High-Fidelity DNA polymerase, Universal PCR primers, and Index (X) Primer. Finally, the PCR products were purified (AMPure XP system), and library quality was assessed using an Agilent Bioanalyzer 2100 system. Clustering of the index-coded samples was performed on a cBot Cluster Generation System using the TruSeq PE Cluster Kit v4-cBot-HS (Illumina) according to the manufacturer’s instructions. After cluster generation, the library preparations were sequenced on an Illumina platform, and paired-end reads were generated.

### 2.6. RNA-Seq Data Analysis

Gene expression levels were estimated using fragments per kilobase per million (FPKM) mapped fragments. Differential expression analyses of the two conditions/groups were performed using DESeq2 and edgeR. The resulting *p*-values were adjusted using Benjamini and Hochberg’s approach to control for the false discovery rate. The FDR < 0.05 and Fold Change ≥ 2 were set as the threshold for significantly differential expression.

### 2.7. Functional Annotation of DEGs

Gene Ontology (GO) enrichment analysis was performed using the GOseq R packages based on Wallenius’ noncentral hypergeometric distribution [31]. GO bubble chart version number: ggplot2, 1.0.1; clusterProfiler, ‘2.0.1’ was used. KEGG enrichment analysis was performed to detect the statistical enrichment degree of differentially expressed genes (DEGs) in the KEGG pathway by clusterProfiler, version number is 4.4.4, and database is KOBAS [32].

### 2.8. Screening Important Candidate Genes

Construction of a gene co-expression network was achieved using Weighted Gene Co-expression Network Analysis (WGCNA) [33]. The expression threshold was set to 1, the module similarity threshold was set to 0.25, and the minimum number of genes in the module was set to 25. The serum biochemical indices of Wujin pigs were co-expressed with all the genes of the WGCNA results. The co-expressed genes were compared with the annotation results of differential gene analysis to obtain the core genes related to the hematopoietic differences between Wujin and Duroc pigs.

### 2.9. Validation of Important Candidate Genes

To verify the RNA-seq results, we performed quantitative reverse transcription polymerase chain reaction (RT-qPCR) on important candidate genes to determine their expression levels in the different groups. rRNAs were reverse-transcribed to cDNAs using the SPARKscript II All-in-one RT SuperMix for qPCR Reagent Kit (Shandong Sparkjade Biotechnology Co., Ltd.). Primers used for RT-qPCR are listed in Table 2. RT-qPCR was performed using 2× SYBR Green qPCR Mix (Shandong Sparkjade Biotechnology Co., Ltd.) in 20 μL volume. RT-qPCR was performed on a QuantStudio 3 Real-Time PCR System (Thermo Fisher Scientific, Shanghai, China) with actin beta (ACTB) as the endogenous control gene, and the relative expression levels were calculated using the 2^−∆∆Ct^ method.

### 2.10. Statistical Analysis

All statistical analyses were performed using SPSS v21.0, with data analyzed using one-way analysis of variance. Differences were considered significant at *p* ≤ 0.05. The results are expressed as the mean ± standard deviation.

## 3. Results

### 3.1. Blood Routine Examination

In Table 3, The Hb content and Mean Corpuscular Hemoglobin Concentration (MCHC) of WGB were significantly higher than those of DGB (*p* < 0.05); the number of platelets was significantly lower than that of DGB (*p* < 0.05); the red blood cells were higher than those of DGB, but the difference was not significant (*p* < 0.05); and the contents of white blood cells and lymphocytes were lower than those of DGB, but the difference was not significant (*p* > 0.05).

### 3.2. Serum Biochemical Indicators

As shown in Table 4, the serum Hb content of WGB was significantly lower than that of DGB (*p* < 0.05), whereas the serum GSH-PX activity and MDA content were higher than those of DGB (*p* > 0.05).

### 3.3. Sequencing Results and Quality Control

**Figure 1:**
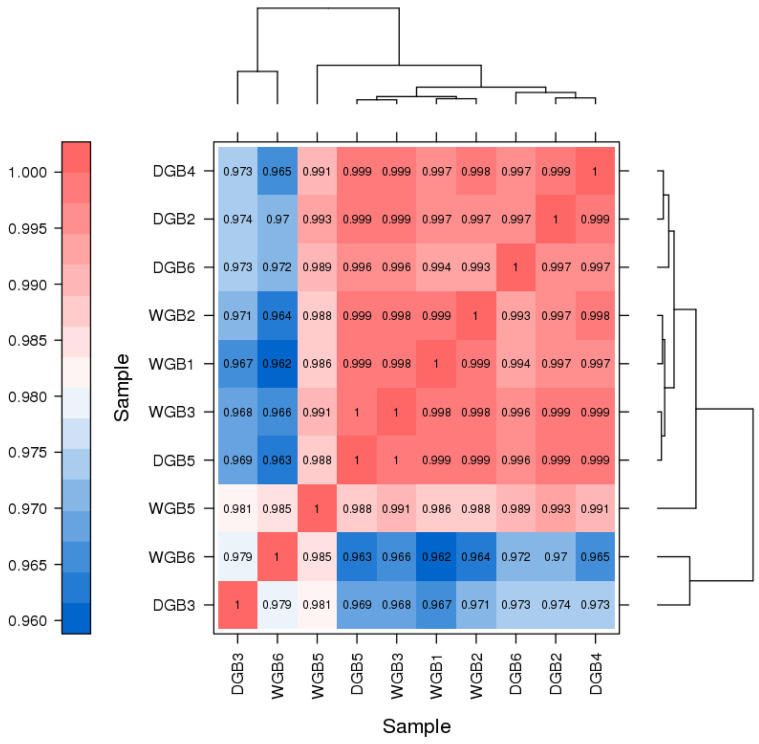
Heat map of expression correlation between two samples. The order of sample size is based on the clustering results related to the samples. The clustering trees corresponding to the samples are displayed on the top and right sides of the figure. The color reflects the correlation between samples.

Five bone marrow tissue samples were sent to Beijing Biomarker Technology Company Co., Ltd. for transcriptome sequencing (Table 5). A total of 79.09 Gb of clean data were obtained, the clean data of each sample reached 5.76 Gb, and the percentage of Q30 bases was 94.64% and above. The clean reads of each sample were sequenced against the designated reference genome, and the matching efficiencies ranged from 96.81% to 97.73%. Based on the comparison results, variable splicing prediction analysis, gene structure optimization analysis, and new gene discovery were performed. A total of 4723 new genes were identified, of which 1031 were functionally annotated. The Pearson correlation coefficient was used to test the correlation between the samples, which showed that the r values were all higher than 95% (Figure 1).

### 3.4. Analysis of Differentially Expressed Genes

**Figure 2:**
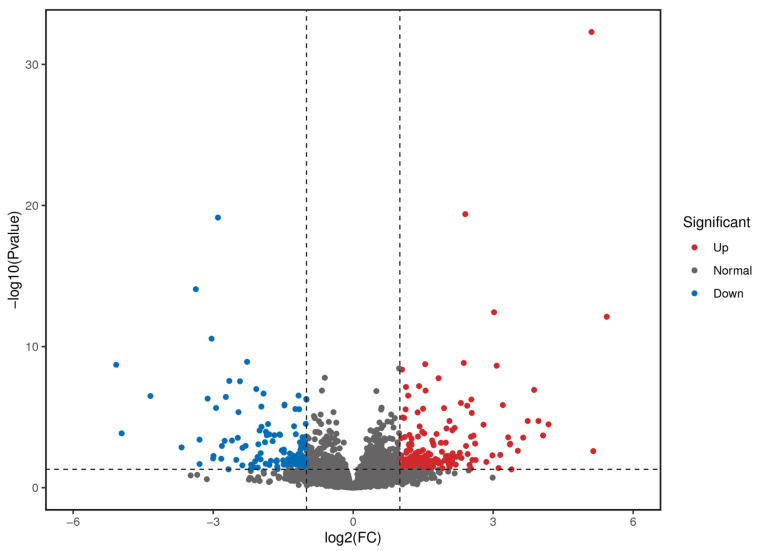
Volcano map of differentially expressed genes. The two vertical dashed lines represent a two-fold expression difference threshold, and the horizontal dashed line corresponds to a *p*-value of 0.05 threshold.

**Figure 3:**
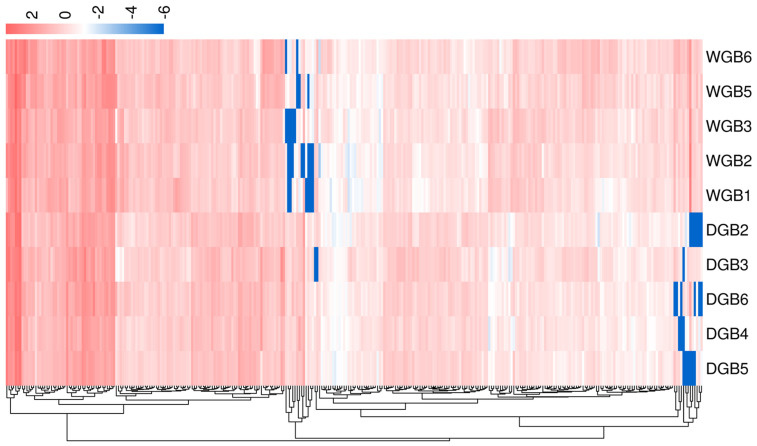
Hierarchical clustering analysis of differentially expressed genes. The abscissa (*x*-axis) represents the sample names and their clustering results, whereas the ordinate (*y*-axis) represents the differentially expressed genes and their clustering results. The color indicates the gene expression levels in the sample, shown as log10 values.

As shown in Figure 2 and Figure 3, 312 significantly differentially expressed genes were identified in Wujin and Duroc pigs, of which 128 were upregulated and 184 were downregulated. Among them, the relative expression of genes in Wujin pigs, including Fc epsilon receptor II (*FCER2*), cluster of differentiation 19 (*CD19*), membrane-spanning 4-domains A1 (*MS4A1*), and leucine-rich repeat-containing 17 (*LRRC17*) showed an upward trend compared with that in Duroc pigs, whereas the relative expression of *LOC106504983* and interleukin 1 beta (*IL1B*) showed a downward trend compared with that in Duroc pigs. These genes may be associated with hematopoietic function.

**Figure 4:**
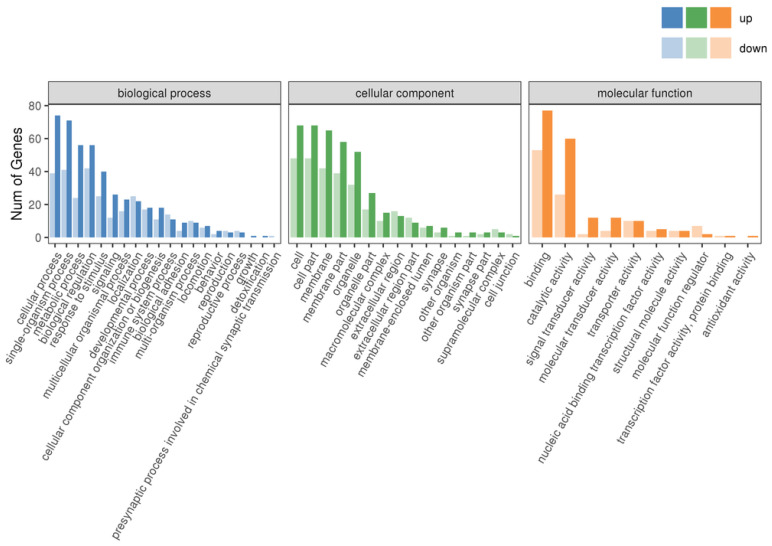
Classification statistics of GO secondary node annotations for differentially expressed genes.

**Figure 5:**
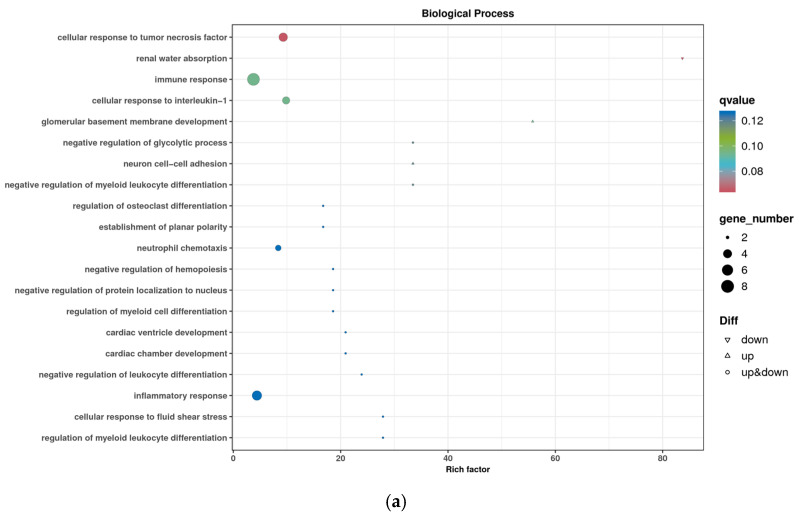

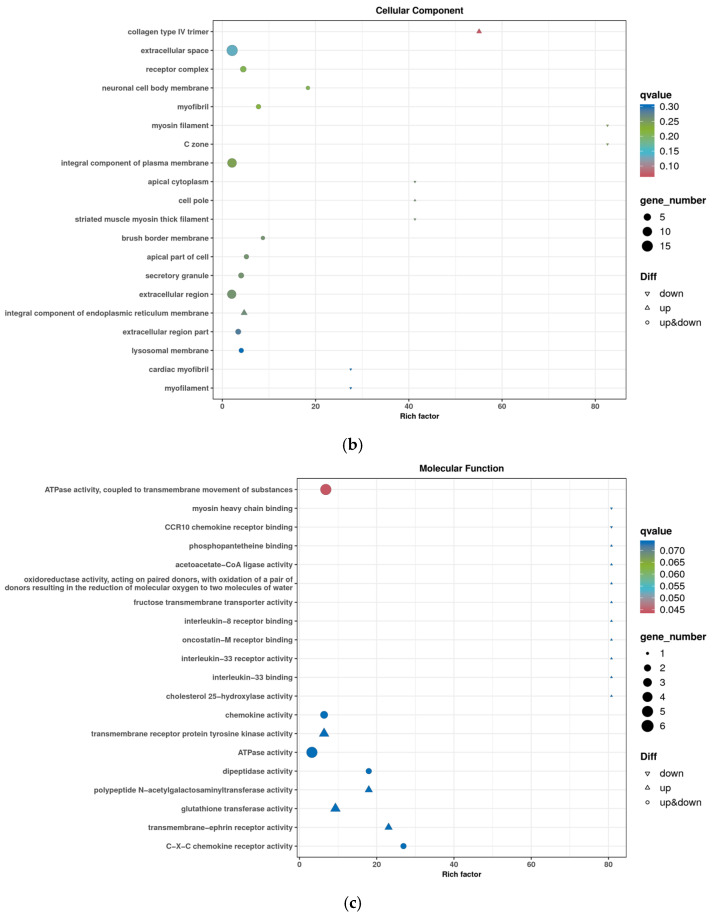
Bubble diagram of GO enrichment. (**a**) Biological Process GO enrichment plot; (**b**) Cellular Component GO enrichment dotplot; (**c**) Molecular Function GO enrichment dotplot.

The classification statistics of the differentially expressed genes were performed at the secondary classification level of the GO database; the results of the GO classification of the differentially expressed genes are shown in Figure 4. The number of genes annotated to the set of differentially expressed genes was 237, and the differential genes were categorized into 58 entries. The top 20 GO classification entries with the most significant DEG enrichment of differential genes in Biological Process (BP), Cell Component (CC), and Molecular Function (MF) are shown in Figure 5. DEGs were significantly enriched in biological processes related to hematopoiesis, inflammation, immunity, and cardiac development. These processes included immune response, cellular response to IL-1, negative regulation of myeloid leukocyte differentiation, regulation of osteoclast differentiation, neutrophil chemotaxis, negative regulation of hemopoiesis, regulation of myeloid cell differentiation, negative regulation of leukocyte differentiation, inflammatory response, regulation of myeloid leukocyte differentiation, C-C chemokine receptor type 10 (CCR10) chemokine receptor binding, IL-8 receptor binding, IL-33 receptor activity, IL-33 binding, glutathione transferase activity, and C-X-C chemokine receptor activity, among others.

**Figure 6:**
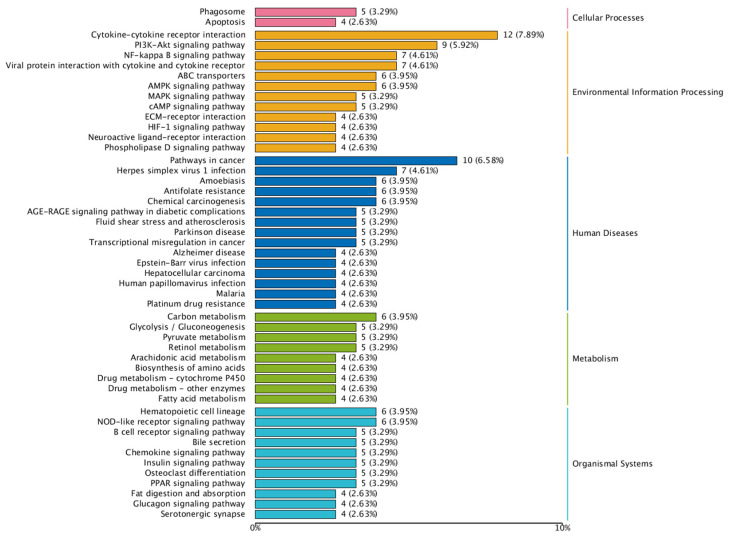
Detailed statistics of KEGG classification of differentially expressed genes.

**Figure 7:**
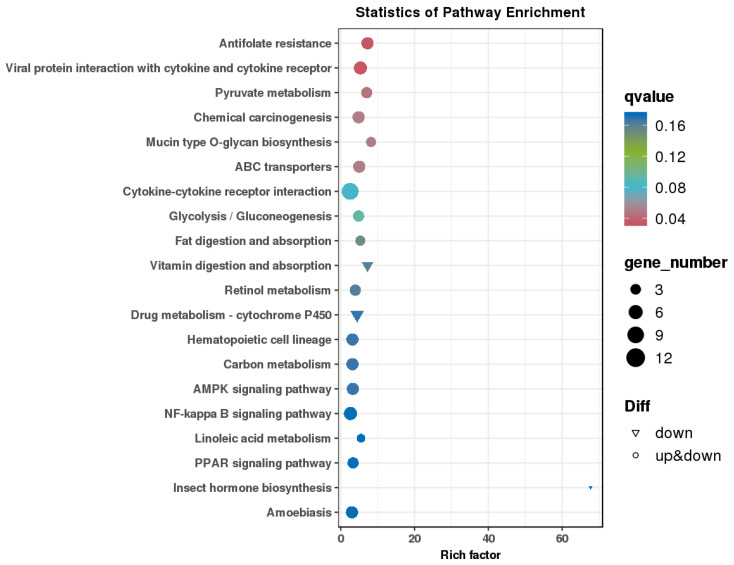
Differential gene KEGG enrichment bubble map.

The number of genes annotated in the KEGG enrichment analysis of the DEG sets was 233, and the differential genes were significantly enriched in 225 KEGG pathways. The results of the KEGG annotation of differentially expressed genes were categorized according to the type of pathway in KEGG; the results are shown in Figure 6. A hypergeometric test was applied to identify the significantly enriched pathways in differentially expressed genes compared with the whole genomic background. The results of KEGG pathway enrichment analysis of differentially expressed genes are shown in Figure 7. The differentially expressed genes were significantly enriched in functions and pathways related to hematopoiesis, inflammation, immunity, and oxidation. These include the AMP-activated protein kinase signaling pathway, nuclear factor-kappa B (NF-κB) signaling pathway, cytokine–cytokine receptor interaction, peroxisome proliferator-activated receptor signaling pathway, and hematopoietic cell lineage, among others.

### 3.5. WGCNA Results

**Figure 8:**
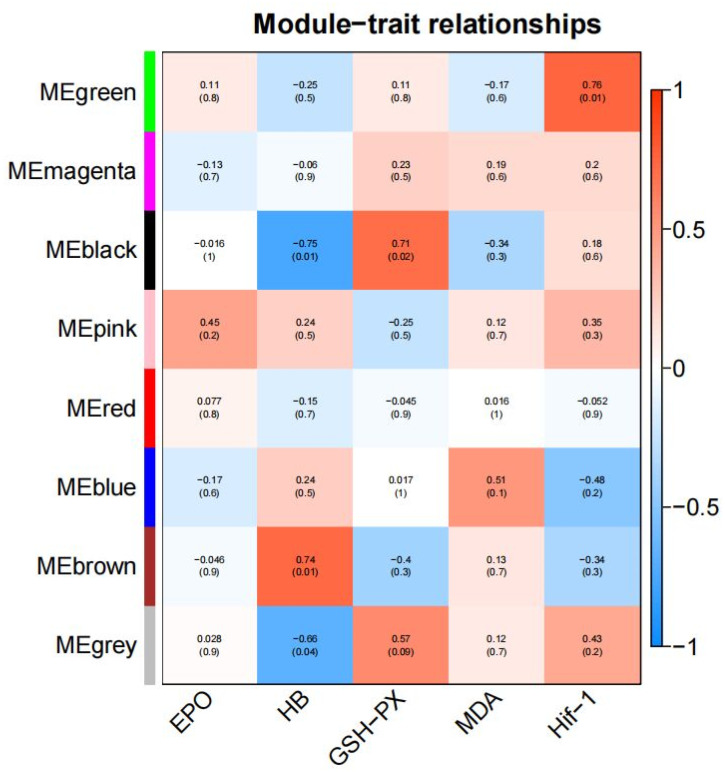
Heat map of module-trait correlations. The modules, represented by different colors on the left, contain a group of co-expressed genes. The first row of data indicates the correlation between a trait and a module, with the numbers in brackets representing the significance of the results.

A gene co-expression network of all genes with serum biochemical indicators was constructed using WGCNA. Correlation heat maps were generated based on module color and serum biochemical indicators; the results are shown in Figure 8. The figure shows that serum EPO level had the highest correlation with the co-expressed genes in the MEpink module (|r| = 0.45, *p* = 0.2), while serum Hb and GSH-PX levels were highly correlated with the co-expressed genes in the MEblack module (HB: |r| = 0.75, *p* = 0.01; GSH-PX: |r| = 0.71, *p* = 0.02).

**Figure 9:**
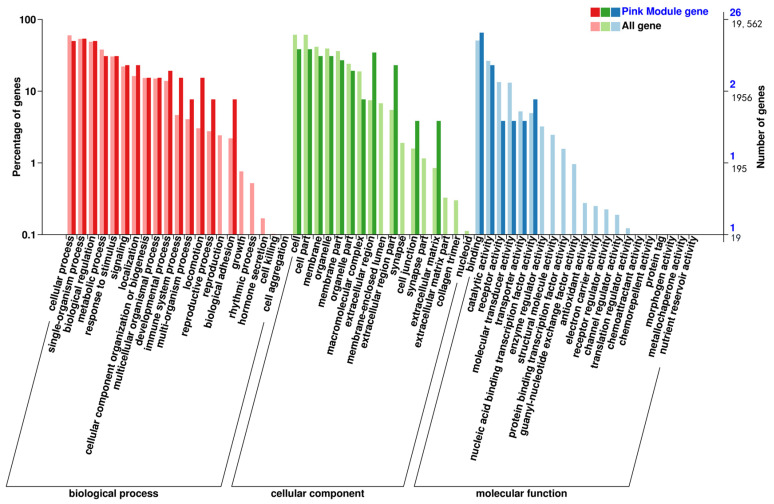
MEpink-enriched GO entry annotation categorization statistical chart.

**Figure 10:**
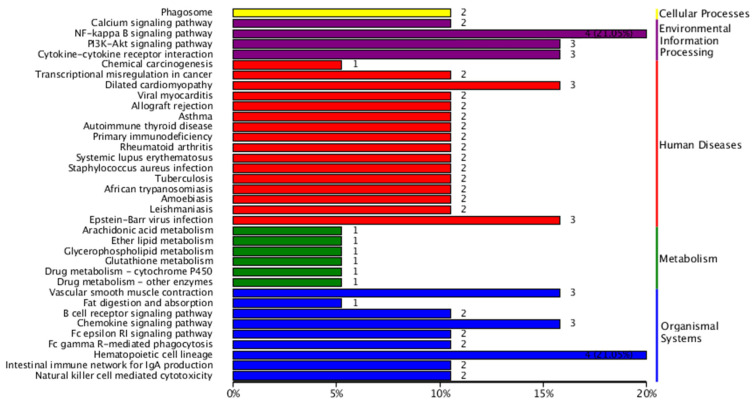
MEpink-enriched KEGG pathway annotation categorization statistics.

The screened gene modules were analyzed using GO functional annotation and KEGG pathway enrichment analyses. The pink module co-expressed genes enriched in GO entries related to hematopoiesis and cardiac development and the KEGG pathway. The GO enrichment results are shown in Figure 9, and the KEGG enrichment results are shown in Figure 10. GO enrichment for hematopoiesis included processes such as ROS metabolic process, osteoblast differentiation, and positive regulation of the bone morphogenetic protein signaling pathway. KEGG enrichment for hematopoiesis involved pathways such as hematopoietic cell lineage, phosphoinositide 3-kinase (PI3K)–protein kinase B (Akt) signaling pathway, and NF-κB signaling pathway.

**Figure 11:**
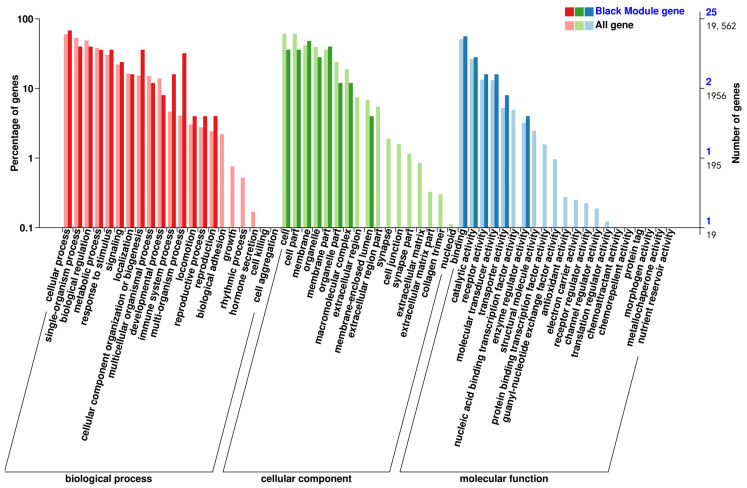
MEblack-enriched GO entry annotation categorization statistical chart.

**Figure 12:**
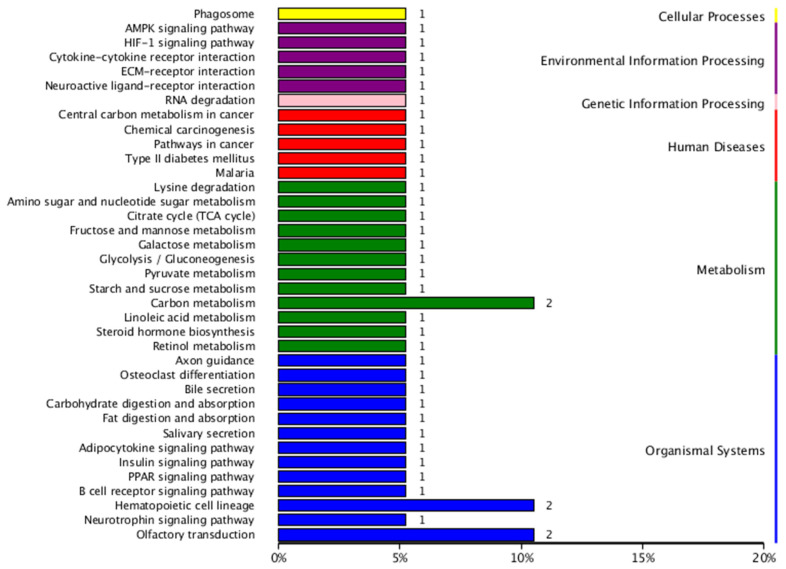
MEblack-enriched KEGG pathway annotation categorization statistics.

The black module co-expressed genes enriched in GO entries related to hematopoiesis and oxidation and the KEGG pathway. The GO enrichment results are shown in Figure 11 and the KEGG enrichment results are shown in Figure 12. GO enrichment for hematopoiesis included processes such as regulation of hemopoiesis, negative regulation of myeloid leukocyte differentiation, regulation of myeloid cell differentiation, negative regulation of myeloid cell differentiation, positive regulation of hemopoiesis, negative regulation of hemopoiesis, and negative regulation of the ROS metabolic process. KEGG enrichment for hematopoietic-related key pathways included the Janus kinase–signal transducer and activator of transcription (JAK-STAT) and *HIF-1* signaling pathways.

### 3.6. Screening and Relative Expression of Core Genes

**Figure 13:**
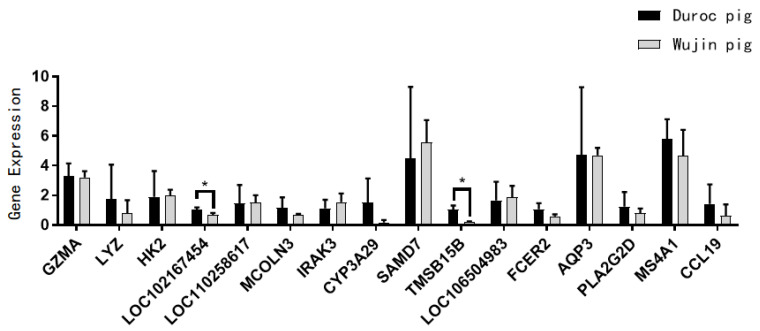
RT-qPCR results after removing unannotated genes. * *p*-values < 0.05.

The differential genes were combined with the WGCNA results to further screen the genes and identify genes whose differential gene sets were the same as those in the two modules, MEpink and MEblack, and a total of 27 genes were obtained. The new genes without annotation were removed, and 16 genes were examined by RT-qPCR; the results are shown in Figure 13. The relative expression of genes in Wujin pigs, including granzyme A (*GZMA*), lysozyme (*LYZ*), *LOC102167454*, mucolipin 3 (*MCOLN3*), cytochrome P450 family 3 subfamily A member 29 (*CYP3A29*), thymosin beta-15B (*TMSB15B*), *FCER2*, aquaporin 3 (*AQP3*), phospholipase A2 group IVD (*PLA2G2D*), *MS4A1,* and *CCL19* showed an upward trend compared with that in Duroc pigs, whereas the relative expression of hexokinase 2 (*HK2*), *LOC110258617*, interleukin-1 receptor-associated kinase 3 (*IRAK3*), sterile alpha motif domain containing 7 (*SAMD7*), and *LOC106504983* showed a downward trend compared with that in Duroc pigs. The RT-qPCR results were consistent with the trend shown by the transcriptome sequencing results. The differential expression of the core genes is essentially consistent with the transcriptome sequencing results. This illustrates the reliability of the sequencing data and candidate core genes screened in this study.

## 4. Discussion

### 4.1. Differences in Blood Physiological and Biochemical Indices Between Wujin and Duroc Pigs

Routine blood tests are common clinical laboratory tests. In this study, the MCHC was significantly higher in Wujin pigs than in Duroc pigs, the number of erythrocytes with mean Hb content was higher than in Duroc pigs, the mean erythrocyte volume was lower than in Duroc pigs, and the number of platelets was significantly lower than in Duroc pigs. The MCHC is a hematological test used to assess the average Hb content of erythrocytes in the blood. Elevated MCHC levels may be associated with various disease-related and non-disease-related factors. Residence in highland areas may lead to elevated levels of MCHC in healthy conditions [34]. The relationship between platelet depletion and highland areas is not direct; however, people who live or work in highland areas may experience physiological changes that affect their platelet number and function.

Pigs living at high altitudes may experience tissue hypoxia. Swine bodies enhance oxygen transport and utilization through several physiological and molecular mechanisms to adapt to this low-oxygen environment. Prolonged exposure to hypoxia can lead to alterations in certain signaling pathways involved in processes such as erythropoiesis, oxygen transport, and energy metabolism, including the JAK/STAT, PI3K/AKT, and *HIF-1* pathways [35]. This study determined the serum levels of *HIF-1*, GSH-PX, and MDA; they are biomarkers associated with the state of oxidative stress in cells and are important for assessing the cellular response to oxidative damage and the antioxidant defense capacity of cells. Among them, serum *HIF-1* levels were lower and serum GSH-PX and MDA levels were higher in Wujin pigs than in Duroc pigs.

*HIF-1* plays an important role in the adaptive response of the organism [36], and its central role is to participate in the hypoxic and hypoxia-adaptive responses of tissue cells [37]. When the body is exposed to hypoxia, *HIF-1* stimulates the kidneys to produce more EPO, which promotes the production of erythrocytes in the bone marrow, thereby increasing the concentration of Hb in the blood to maintain normal oxygen transport and delivery [38]. Therefore, lower serum levels of *HIF-1* may indicate that Wujin pigs are better adapted to low-oxygen environments than Duroc pigs. In a hypoxic environment, the body may produce more erythrocytes to increase its oxygen-carrying capacity [39]. In the present study, there was no significant difference in serum EPO levels between Wujin and Duroc pigs; therefore, the difference in erythrocytes between Wujin and Duroc pigs may be due to the difference in erythropoietin receptors, which needs to be further investigated. In this study, the serum Hb levels of Wujin pigs were significantly lower than those of Duroc pigs. Normally, serum Hb levels are very low as Hb is mainly present in erythrocytes. However, the average Hb level in Wujin pigs was higher than that in Duroc pigs in routine blood tests, potentially due to Hb being mainly present in erythrocytes, and while the number of erythrocytes was not significantly different, this suggests that Wujin pigs have a higher content of Hb in erythrocytes and a higher oxygen-carrying capacity than Duroc pigs.

Elevated serum levels of GSH-PX and MDA usually indicate that the organism has suffered oxidative damage. GSH-PX is an important antioxidant enzyme that catalyzes the reaction of reduced glutathione with hydrogen peroxide or lipid peroxides (e.g., ROOH) to protect cells from oxidative damage [40]. MDA is one of the major products of lipid peroxidation and is often used as a biomarker of oxidative stress. Elevated MDA levels usually indicate increased peroxidation of cell membrane lipids, which is a sign of oxidative damage to cells. Oxidative damage is mainly caused by the overproduction of ROS and reactive nitrogen or by deficiencies in the antioxidant defense system [41]. These reactive substances are by-products of cellular metabolism under normal physiological conditions, and at certain concentrations, are necessary for cell signaling and normal functioning of the organism [42]. Oxygen is a substrate for ROS production, and an increase in oxygen levels in the body can lead to an increase in ROS in the body [43]. However, when ROS production exceeds the cell’s ability to scavenge ROS, it can lead to an increased risk of oxidative damage, resulting in oxidative stress. The blood of Wujin pigs contains higher Hb and erythrocyte counts, which may result in more oxygen in the organism and higher levels of ROS in the body than in Duroc pigs. In this study, serum GSH-PX and MDA levels were higher in Wujin pigs than in Duroc pigs, suggesting that the antioxidant mechanism was activated in Wujin pigs and that the cells were more capable of scavenging oxidants. In contrast, Dong [44] showed that an increase in ROS leads to platelet hyperactivation, which increases the platelet count and the risk of thrombosis. The results of this study showed that the blood platelet count of Wujin pigs was significantly lower than that of Duroc pigs, indicating that Wujin pigs could effectively regulate the amount of ROS in the body, and their antioxidant capacity was higher than that of Duroc pigs. Although low ROS levels are essential to maintain HSC quiescence, elevated ROS levels are necessary for HSC transformation [45]. Under excessive oxidative stress, stem cell function is impaired, and HSCs tend to undergo myeloid differentiation [46,47]. High ROS levels ultimately lead to HSC failure and apoptosis [48]. Therefore, the high antioxidant capacity of Wujin pigs ensures the stability of ROS levels in the organism, ensuring HSC differentiation.

### 4.2. Transcriptomic Analysis

The GO enrichment results showed that the differentially expressed genes were significantly enriched in biological processes related to hematopoiesis, inflammation, immunity, heart development, cellular components related to muscle cells, and molecular functions related to inflammation and oxidation. The modes of regulating hematopoiesis, inflammation, and oxidative stress in organisms form a complex network that maintains internal environmental stability [49,50,51]. The effects of inflammation and oxidative stress on the hematopoietic system are primarily reflected in their effects on HSCs and progenitor cells. Systemic inflammation and oxidative stress affect the hematopoietic microenvironment of the bone marrow [52,53], directly damaging HSCs and affecting their self-renewal and differentiation capacity [54,55,56]. The results of differential gene GO enrichment suggested differences in the aspects of organismal immunity and inflammation between Wujin and Duroc pigs, implying that the differences in hematopoietic mechanisms may be mainly due to differences in antioxidant capacity. Antioxidant capacity affects hematopoiesis by influencing immune and inflammatory mechanisms, and indirectly regulating angiogenesis, cardiogenesis, and osteogenesis in Wujin pigs.

The KEGG enrichment results showed that the differentially expressed genes were highly enriched in pathways related to life activities. Among the many enriched pathways, the most important pathways directly related to the hematopoietic process are the hematopoietic cell lineage and signaling pathways that regulate stem cell pluripotency, essential for the maintenance and differentiation of HSCs [57]. The JAK-STAT signaling pathway, as a pathway affecting the signaling pathways regulating stem cell pluripotency, plays a key role in the proliferation and differentiation of multiple cell types. This pathway regulates hematopoietic cell function and the hematopoietic microenvironment by modulating multiple hematopoietic growth factors and directly and indirectly regulates HSCs, hematopoiesis, and development [58,59]. Pathways directly related to inflammation, immunity, and other processes that indirectly affect the hematopoietic function of the organism were also enriched, such as the NF-κB pathway, IL-17 pathway, chemokine pathway, and primary immunodeficiency. These pathways are directly or indirectly involved in the hematopoietic process and affect the generation, development, and function of blood cells [60,61,62]. The KEGG enrichment results also revealed differences in immunity and inflammation between Wujin and Duroc pigs, which directly and indirectly affect hematopoiesis.

The GO enrichment results obtained using WGCNA for the pink and black modules were similar to the differential gene enrichment results, and the co-expressed genes were mainly enriched in GO entries related to hematopoiesis, oxidation, inflammation, and bone formation. It can also be indicated that the difference in hematopoiesis between Wujin and Duroc pigs is due to their antioxidant ability. JAK-STAT, PI3K-Akt, NF-κB, and HIF-1 are intracellular signaling pathways that play important roles in hematopoiesis. At the same time, crosstalk exists between them, which can regulate and influence each other, forming a regulatory network in the organism [63,64,65]. The body’s environment is regulated by cytokine production, cell proliferation and differentiation, and cell survival and migration, which in turn maintain and regulate the hematopoietic function and homeostasis of the organism.

### 4.3. Key Differential Genes for Hematopoiesis in Wujin and Duroc Pigs

RNA sequencing results revealed that six genes directly affected hematopoiesis. Among them, the upregulation of *FCER2* gene expression may indirectly affect the self-renewal and differentiation capacities of HSCs, as well as the maturation and function of blood cells [66]. The upregulation of *FCER2* gene expression may also promote the proliferation and differentiation of B cells [67], increase the production of immunoglobulin E (IgE) [68], promote the uptake and presentation of IgE-dependent antigens to T cells, and affect the initiation and regulation of immune responses, thereby affecting hematopoiesis. The *CD19* gene may affect the function of HSCs, thereby affecting the normal hematopoietic process. In patients with acute lymphoblastic leukemia, HSC transplantation following CD19 chimeric antigen receptor T therapy can improve therapeutic efficacy [69]. Additionally, *CD19* regulates the development and function of B cells, and the upregulation of its expression can affect the development, proliferation, and differentiation of B cells, as well as mechanisms related to inflammatory responses and immune cell activation [69]. *MS4A1,* mainly expressed in B cells, plays an important role in immunity. Studies have shown that members of the MS4A family are expressed in different types of white blood cells, potentially contributing to their differentiation and function [70]. The upregulation of *MS4A1* gene expression may promote the proliferation and differentiation of B cells and affect the activation and signal transduction of immune cells. *LRRC17* can control mitochondrial autophagy in bone marrow mesenchymal stem cells and affect their aging process. The upregulation of *LRRC17* gene expression can regulate the self-renewal and differentiation abilities of HSCs by affecting mitochondrial autophagy, facilitating the maintenance of their young state and function. This process promotes the renewal of HSCs and subsequently affects the hematopoietic process [71]. Notably, the downregulation of *IL1B* gene expression may have various effects on hematopoiesis. *IL1B* is an inflammatory factor that regulates the function of HSCs [72]. Chronic exposure to *IL1B* causes HSCs to differentiate prematurely into bone marrow at the expense of their self-renewal capacity [73]. *IL1B* can also participate in the regulation of immune responses, influencing the body’s inflammatory response and affecting the microenvironment of HSCs [74]. The downregulation of *IL1B* gene expression may reduce the inflammatory stimulation to HSCs, facilitating the maintenance of HSC homeostasis, reducing the shift of HSCs toward the myeloid system, and enhancing their self-renewal ability.

The results of WGCNA showed that the core DEGs were primarily involved in functions related to immunity, inflammation, and apoptosis induction. Among them, *GZMA* can affect the generation of human umbilical vein endothelial cells; therefore, it may play a role in regulating angiogenesis [75]. Not only is *GZMA* able to induce apoptosis but it may also lead to cell death through other mechanisms, indicating that the diverse roles of *GZMA* in the immune response provide important information [76,77,78]. *HK2* is mainly localized to the outer mitochondrial membrane and plays a role in a variety of biological processes, including energy metabolism, cell proliferation, and immune responses. It may affect the homeostatic environment of myeloid cells by regulating metabolism, which in turn affects hematopoiesis [79,80]. *MCOLN3*, by regulating the intracellular levels of calcium ions, affects the formation of autophagosomes and their fusion with lysosomes, which in turn affects the efficiency of autophagy [81,82]. The activity of *LYZ* is important for modulating inflammatory responses and promoting apoptosis and immune responses [83]. Qin et al. [84] constructed a comprehensive cellular map as a reference for hematopoiesis and used *LYZ* as a marker gene for HSCs. Because autophagy plays a key role in the maintenance and differentiation of HSCs, *MCOLN3* and *LYZ* may indirectly affect the hematopoietic process by regulating autophagy. *CCL19* can induce anti-tumor immune responses and inhibit angiogenesis, exerting tumor suppressor functions, and also induce inflammation, cell growth, and metastasis [85]. Unfortunately, due to the transcriptome sequencing results of new genes and less-studied genes, we cannot discuss all the core gene functions. However, according to the current results, the hematopoietic differences between the Wujin and Duroc pigs come mainly from their differences in immunity and inflammation, suggesting that Wujin pigs have a better antioxidant capacity and disease resistance, which can regulate the hematopoietic capacity of the organism.

## 5. Conclusions

Oxygen transport capacity, antioxidant capacity, and hypoxia adaptation were greater in Wujin pigs than in Duroc pigs; these mechanisms resulted in a greater hematopoietic capacity in Wujin pigs. The transcriptomic analysis yielded a total of 312 significant differential genes, among which *FCER2*, *CD19*, *MS4A1*, *LOC106504983 LRRC17*, and *IL1B* may be related to hematopoiesis. The results of differential gene GO and KEGG enrichment analyses indicated differences in immune, inflammatory, and oxidative stress capacities between Wujin and Duroc pigs; these mechanisms could indirectly regulate the hematopoietic capacity of Wujin pigs. Sixteen core genes were screened for their association with serum EPO and Hb levels in Wujin pigs, namely *GZMA*, *LYZ*, *HK2*, *LOC102167454*, *LOC110258617*, *MCOLN3*, *IRAK3*, *CYP3A29*, *SAMD7*, *TMSB15B*, *LOC106504983*, *FCER2*, *AQP3*, *PLA2G2D*, *MS4A1*, and *CCL19*. These genes may be candidates for studying hematopoietic differences between Wujin and Duroc pigs.

## Figures and Tables

**Table 1:** Diet formula and nutrition level.

Ingredient	Content (%)	Nutrient Level	Content
Corn	30.0	CP (%)	19.0
Soybean meal	15.0	Ca (%)	0.80
Expanded soybean	15.0	Fe * (%)	0.02
Whey powder	12.0	AP (%)	0.42
Rice	10.0	Lys (%)	1.22
Flour	6.0	Met (%)	0.39
Glucose	4.0	Methionine + cystine (%)	0.67
Peruvian fish meal	3.0	Thr (%)	0.77
Citric acid	1.50	Trp (%)	0.23
Vegetable oil	1.00	DE (MJ/kg)	13.38
Fine stone powder	0.84		
Calcium hydrogen phosphate	0.81		
Lysine	0.18		
Fungicide	0.10		
DL-methionine	0.08		
Premix	0.49		

Premix provides the following nutrients (per kg): VA, 100 mg; VD3, 100 mg; VE, 4400 mg; VK, 100 mg; VB1, 100 mg; VB2, 300 mg; Cu, 10 mg; Zn, 110 mg; Mn, 30 mg; I, 0.2 mg; Se, 0.3 mg; VB3, 1000 mg; VB5, 800 mg; VB12, 500 mg; biotin, 1500 mg; folic acid, 100 mg; and zeolite powder, 298.73 g. *: Fe was calculated, other items were measured.

**Table 2:** PCR primer sequences.

Gene ID	Primer Sequence (5′-3′)	Amplification Length/bp
*LYZ*	F:GCAAGACACCCAAAGCAGTT	178
	R:CCCCGAATGTACTGCGAGAC	
*HK2*	F:CCTTGGCCCGCTGAGATAAA	117
	R:CCACGGGGATTAGCAAAGGT	
*LOC102167454*	F:CAGGAGGGGAAGGACAAACG	188
	R:CGGGCTAAGGGTTGTCTTGA	
*LOC110258617*	F:CCGTAGCAGGAAATGTGGCT	224
	R:TTCACTAGAGCAGTTATCATCAGCA	
*MCOLN3*	F:GGGGTAGGGAGCAGTTAGGA	198
	R:AGTCGCCAACAGGATGGAAG	
*IRAK3*	F:TCCCACATCCCTGAAGTCCT	230
	R:TGCAGGCATCCTCATTCCTC	
*CYP3A29*	F:AGGACTTGGGCTTTGATGGTC	250
	R:ATACTAGGTGGGGGTGGATGG	
*SAMD7*	F:TGCAACTATGCCGAACATGC	221
	R:TGGGGCCATAGAGGAAAGGT	
*TMSB15B*	F:CCAGCAGGAGAAAGAGTGTGT	165
	R:GCCAGGCATTACAGGTGAGA	
*LOC106504983*	F:GACCCTGAGACGCACACAA	152
	R:TAGAGGTGATGTGCTGGGTG	
*FCER2*	F:CTTTGGTGAGACGGCCAAGA	151
	R:CAGGTCCCGAAGACCAATCC	
*AQP3*	F:CACCTACCCCTCTGGACACT	200
	R:TGACGGCATAGCCAGAGTTG	
*PLA2G2D*	F:CGCAACCCCCAAAGGATCTA	128
	R:CAAGTGGCAGACCCATTCCT	
*MS4A1*	F:ACTGCTCGCTTCCAAACTGA	107
	R:TTCTGGGCGTCGTCATTTCA	
*CCL19*	F:CGCTACCTGCTCATTCGAGA	117
	R:TCTCTTGATGATGCGCTCCAC	
*GZMA*	F:TGACCTGAAAGGCAACCCTC	229
	R:GGTGTGGGGTTCGACATCTT	
*ACTB*	F:GCGGCATCCACGAAACTA	75
	R:TGTTGGCGTAGAGGTCCTT	

**Table 3:** Routine blood parameters of Wujin and Duroc pigs.

Items	DGB	WGB
Leukocyte (×10^9^/L)	17.96 ± 3.28	16.5 ± 4.7
Lymphocyte (×10^9^/L)	8.9 ± 2.46	5.78 ± 3.18
monocytes (×10^9^/L)	1.96 ± 0.27	1.78 ± 1.1
Neutrophil (×10^9^/L)	7.1 ± 1.48	8.9 ± 1.65
Lymphocytes percentage (%)	49.4 ± 6.18	32.83 ± 15.3
Monocyte percentage (%)	10.68 ± 1.04	9.85 ± 4.45
Neutrophils percentage (%)	39.92 ± 6.42	57 ± 18.67
Erythrocyte (×10^12^/L)	7.52 ± 0.8	7.97 ± 1.23
Hemoglobin (g/L)	12.9 ± 1	13.93 ± 1.38 *
Packed cell volume (%)	39.04 ± 3.55	39.85 ± 2.85
Mean corpuscular volume (fL)	51.96 ± 0.96	50.35 ± 3.63
Mean corpuscular hemoglobin (pg)	17.22 ± 0.54	17.6 ± 0.87
Mean corpuscular hemoglobin concentration (g/L)	33.2 ± 0.84	35 ± 0.82 *
Red cell distribution width (CV%)	17.76 ± 0.36	16.83 ± 0.85
Red cell distribution width (SD fL)	37.84 ± 1.35	35.28 ± 4.08
Platelet (×10^9^/L)	274.8 ± 22.6 *	194.75 ± 47.35
MPV (fL)	5.9 ± 0.35	6.1 ± 0.42

*: (*p*-values < 0.05.)

**Table 4:** Serum hematopoietic parameters of Wujin and Duroc pigs.

Items	DGB	WGB
EPO/mIU·mL^−1^	4.90 ± 0.53	4.89 ± 0.23
Hb/μg·mL^−1^	68.64 ± 2.74 *	61.41 ± 1.79
HIF1/pg·mL^−1^	394.5 ± 33.98	374.22 ± 31.04
GSH-PX	860.38 ± 212.72	1005.28 ± 486.88
MDA/nmol·mL^−1^	1.81 ± 0.30	2.34 ± 1.43

*: *p*-values < 0.05.

**Table 5:** Sequencing data statistics and comparison results.

Samples	Clean Reads	Clean Bases	GC Content (%)	% ≥ Q30 (%)	Total Reads	Mapped Reads	Uniq Mapped Reads
DGB2	28,469,394	8,500,924,602	52.98%	94.79%	56,938,788	55,474,814 (97.43%)	47,137,369 (82.79%)
DGB3	22,764,914	6,806,034,552	52.19%	94.89%	45,529,828	44,345,494 (97.40%)	40,143,984 (88.17%)
DGB4	27,326,906	8,165,439,618	53.35%	95.19%	54,653,812	53,293,026 (97.51%)	45,501,090 (83.25%)
DGB5	28,422,180	8,489,993,794	53.89%	95.16%	56,844,360	55,424,264 (97.50%)	46,814,234 (82.36%)
DGB6	19,245,459	5,756,626,694	53.40%	95.24%	38,490,918	37,528,942 (97.50%)	32,209,925 (83.68%)
WGB1	29,770,100	8,889,006,784	54.00%	95.04%	59,540,200	57,897,952 (97.24%)	48,075,857 (80.75%)
WGB2	28,121,773	8,406,190,620	53.39%	94.98%	56,243,546	54,447,710 (96.81%)	46,307,875 (82.33%)
WGB3	27,338,088	8,161,849,630	53.03%	94.82%	54,676,176	53,120,625 (97.15%)	46,529,517 (85.10%)
WGB5	25,699,553	7,686,844,086	52.47%	94.69%	51,399,106	49,937,139 (97.16%)	44,979,012 (87.51%)
WGB6	27,510,543	8,229,104,936	50.65%	94.64%	55,021,086	53,772,021 (97.73%)	50,942,780 (92.59%)

## Data Availability

Raw reads of transcriptome sequencing of the bone marrow are available at NCBI (PRJNA1170427: http://www.ncbi.nlm.nih.gov/bioproject/1170427 (accessed on 1 February 2024)).

## References

[1] Deng M.L., Feng G.Y., Li R., Yin Y.L. (2024). Research Progress on Germplasm Characteristics and Nutrition of Wujin Pig. Chin. J. Anim. Nutr..

[2] Gao L., Xing X., Guo R., Li Q., Xu Y., Pan H., Ji P., Wang P., Yu C., Li J. (2024). Effect of Different Dietary Iron Contents on Liver Transcriptome Characteristics in Wujin Pigs. Animals.

[3] Li M., Zhang C., An Q., Pan H., Chen K., Guo R. (2015). The Study of Hypoxia Adaptive Differences of Yunnan Wujin and Yuedawu Pigs. Acta Vet. Zootech. Sin..

[4] Zhang C., Chen K., Huang J., Guo R. (2012). Glutaredoxin 1 and Thioredoxin 1: Gene Expression Characteristics in Different Tissues and Effects of L-Histidine on Gene Expressions in Oxidant Stress Cells of Yunnan Wujin Pigs. Chin. J. Anim. Nutr..

[5] Hamey F.K., Lau W.W., Kucinski I., Wang X., Diamanti E., Wilson N.K., Göttgens B., Dahlin J.S. (2020). Single-cell molecular profiling provides a high-resolution map of basophil and mast cell development. Allergy.

[6] Cantor A.B., Orkin S.H. (2002). Transcriptional regulation of erythropoiesis: An affair involving multiple partners. Oncogene.

[7] Rhodes J., Hagen A., Hsu K., Deng M., Liu T.X., Look A.T., Kanki J.P. (2005). Interplay of pu. 1 and gata1 determines myelo-erythroid progenitor cell fate in zebrafish. Dev. Cell.

[8] Scott E.W., Simon M.C., Anastasi J., Singh H. (1994). Requirement of transcription factor PU. 1 in the development of multiple hematopoietic lineages. Science.

[9] Brown G., Ceredig R., Tsapogas P. (2018). The Making of Hematopoiesis: Developmental Ancestry and Environmental Nurture. Int. J. Mol. Sci..

[10] Jacobsen S.E., Nerlov C. (2019). Haematopoiesis in the era of advanced single-cell technologies. Nat. Cell Biol..

[11] Ding L., Saunders T.L., Enikolopov G., Morrison S.J. (2012). Endothelial and perivascular cells maintain haematopoietic stem cells. Nature.

[12] Greenbaum A., Hsu Y.M., Day R.B., Schuettpelz L.G., Christopher M.J., Borgerding J.N., Nagasawa T., Link D.C. (2013). CXCL12 in early mesenchymal progenitors is required for haematopoietic stem-cell maintenance. Nature.

[13] Li W., Wang Y., Chen L., An X. (2019). Erythroblast island macrophages: Recent discovery and future perspectives. Blood Sci..

[14] Li W., Guo R., Song Y., Jiang Z. (2021). Erythroblastic island macrophages shape normal erythropoiesis and drive associated disorders in erythroid hematopoietic diseases. Front. Cell Dev. Biol..

[15] Bai J., Fan F., Gao C., Li S., Li W., Wei T., Cheng S., Yu J., Zheng C., Zhao J. (2023). CD169-CD43 interaction is involved in erythroblastic island formation and erythroid differentiation. Haematologica.

[16] Lévesque J.P., Summers K.M., Bisht K., Millard S.M., Winkler I.G., Pettit A.R. (2021). Macrophages form erythropoietic niches and regulate iron homeostasis to adapt erythropoiesis in response to infections and inflammation. Exp. Hematol..

[17] Storz J.F., Bautista N.M. (2022). Altitude acclimatization, hemoglobin-oxygen affinity, and circulatory oxygen transport in hypoxia. Mol. Aspects Med..

[18] Cheng Y., Miller M.J., Zhang D., Xiong Y., Hao Y., Jia C., Cai T., Li S.H., Johansson U.S., Liu Y. (2021). Parallel genomic responses to historical climate change and high elevation in East Asian songbirds. Proc. Natl. Acad. Sci. USA.

[19] Ivy C.M., Wearing O.H., Natarajan C., Schweizer R.M., Gutiérrez-Pinto N., Velotta J.P., Campbell-Staton S.C., Petersen E.E., Fago A., Cheviron Z.A. (2022). Genetic variation in haemoglobin is associated with evolved changes in breathing in high-altitude deer mice. J. Exp. Biol..

[20] Storz J.F. (2016). Hemoglobin–oxygen affinity in high-altitude vertebrates: Is there evidence for an adaptive trend?. J. Exp. Biol..

[21] Ivy C.M., Scott G.R. (2017). Control of breathing and ventilatory acclimatization to hypoxia in deer mice native to high altitudes. Acta Physiol..

[22] Meurens F., Summerfield A., Nauwynck H., Saif L., Gerdts V. (2012). The pig: A model for human infectious diseases. Trends Microbiol..

[23] Swindle M.M., Makin A., Herron A.J., Clubb F.J., Frazier K.S. (2012). Swine as models in biomedical research and toxicology testing. Vet. Pathol..

[24] Li Q., Liang Z., Lu T. (2024). Differentiation Potential Analysis of Bone Marrow Mesenchymal Stem Cells from Guizhou Miniature Pigs. Adv. Eng. Technol. Res..

[25] Gerdts V., Wilson H.L., Meurens F., van Drunen Littel-van den Hurk S., Wilson D., Walker S., Wheler C., Townsend H., Potter A.A. (2015). Large animal models for vaccine development and testing. ILAR J..

[26] Gutierrez K., Dicks N., Glanzner W.G., Agellon L.B., Bordignon V. (2015). Efficacy of the porcine species in biomedical research. Front. Genet..

[27] Klymiuk N., Seeliger F., Bohlooly-Y M., Blutke A., Rudmann D.G., Wolf E. (2016). Tailored pig models for preclinical efficacy and safety testing of targeted therapies. Toxicol. Pathol..

[28] Pabst R. (2020). The pig as a model for immunology research. Cell Tissue Res..

[29] De Lange C.F.M. (2013). New NRC (2012) Nutrient Requirements of Swine. Adv. Pork Prod..

[30] (2020). Nutrient Requirements of Swine.

[31] Young M.D., Wakefield M.J., Smyth G.K., Oshlack A. (2010). Gene ontology analysis for RNA-seq: Accounting for selection bias. Genome Biol..

[32] Mao X., Cai T., Olyarchuk J.G., Wei L. (2005). Automated genome annotation and pathway identification using the KEGG Orthology (KO) as a controlled vocabulary. Bioinformatics.

[33] Langfelder P., Horvath S. (2008). WGCNA: An R Package for Weighted Correlation Network Analysis. BMC Bioinform..

[34] Alkhaldy H.Y., Awan Z.A., Abouzaid A.A., Elbahaey H.M., Al Amoudi S.M., Shehata S.F., Saboor M. (2022). Effect of altitude on hemoglobin and red blood cell indices in adults in different regions of Saudi Arabia. Int. J. Gen. Med..

[35] Wilkes M.C., Shibuya A., Sakamoto K.M. (2021). Signaling pathways that regulate normal and aberrant red blood cell development. Genes.

[36] Wiesener M.S., Jürgensen J.S., Rosenberger C., Scholze C.K., Hörstrup J.H., Warnecke C., Mandriota S., Bechmann I., Frei U.A., Pugh C.W. (2003). Widespread hypoxia-inducible expression of HIF-2alpha in distinct cell populations of different organs. FASEB J..

[37] Bracken C.P., Whitelaw M.L., Peet D.J. (2003). The hypoxia-inducible factors: Key transcriptional regulators of hypoxic responses. Cell. Mol. Life Sci..

[38] Metcalf D. (2008). Hematopoietic cytokines. Blood.

[39] Song J., Sundar K.M., Hoidal J., Prchal J.T. (2019). Hematological changes in chronic sustained hypoxia and chronic intermittent hypoxia in a mouse model. Blood.

[40] Panday S., Talreja R., Kavdia M. (2020). The role of glutathione and glutathione peroxidase in regulating cellular level of reactive oxygen and nitrogen species. Microvasc. Res..

[41] Aranda-Rivera A.K., Cruz-Gregorio A., Arancibia-Hernández Y.L., Hernández-Cruz E.Y., Pedraza-Chaverri J. (2022). RONS and Oxidative Stress: An Overview of Basic Concepts. Oxygen.

[42] Shivakumar A., Yogendra Kumar M.S. (2018). Critical review on the analytical mechanistic steps in the evaluation of antioxidant activity. Crit. Rev. Anal. Chem..

[43] Sies H., Jones D.P. (2020). Reactive oxygen species (ROS) as pleiotropic physiological signalling agents. Nat. Rev. Mol. Cell Biol..

[44] Dong H., Li H., Fang L., Zhang A., Liu X., Xue F., Chen Y., Liu W., Chi Y., Wang W. (2023). Increased reactive oxygen species lead to overactivation of platelets in essential thrombocythemia. Thromb. Res..

[45] Ye Z.W., Zhang J., Townsend D.M., Tew K.D. (2015). Oxidative stress, redox regulation and diseases of cellular differentiation. BBA-Gen. Subj..

[46] Ito K., Hirao A., Arai F., Takubo K., Matsuoka S., Miyamoto K., Ohmura M., Naka K., Hosokawa K., Ikeda Y. (2006). Reactive oxygen species act through p38 MAPK to limit the lifespan of hematopoietic stem cells. Nat. Med..

[47] Jang Y.Y., Sharkis S.J. (2007). A low level of reactive oxygen species selects for primitive hematopoietic stem cells that may reside in the low-oxygenic niche. Blood.

[48] Ito K., Hirao A., Arai F., Matsuoka S., Takubo K., Hamaguchi I., Nomiyama K., Hosokawa K., Sakurada K., Nakagata N. (2004). Regulation of oxidative stress by ATM is required for self-renewal of haematopoietic stem cells. Nature.

[49] Anderson G.A., Rodriguez M., Kathrein K.L. (2020). Regulation of stress-induced hematopoiesis. Curr. Opin. Hematol..

[50] Salama R.M., Abbas S.S., Darwish S.F., Sallam A.A., Elmongy N.F., El Wakeel S.A. (2023). Regulation of NOX/p38 MAPK/PPARα pathways and miR-155 expression by boswellic acids reduces hepatic injury in experimentally-induced alcoholic liver disease mouse model: Novel mechanistic insight. Arch. Pharm. Res..

[51] Bousounis P., Bergo V., Trompouki E. (2021). Inflammation, aging and hematopoiesis: A complex relationship. Cells.

[52] Xu Y., Murphy A.J., Fleetwood A.J. (2022). Hematopoietic progenitors and the bone marrow niche shape the inflammatory response and contribute to chronic disease. Int. J. Mol. Sci..

[53] Ho N.P.Y., Takizawa H. (2022). Inflammation regulates haematopoietic stem cells and their niche. Int. J. Mol. Sci..

[54] Leimkühler N.B., Schneider R.K. (2019). Inflammatory bone marrow microenvironment. Hematol. ASH Educ. Program.

[55] Ali M.A., Park C.Y. (2020). A new view of hematopoiesis during inflammation. Blood.

[56] Hosokawa K., Arai F., Yoshihara H., Nakamura Y., Gomei Y., Iwasaki H., Miyamoto K., Shima H., Ito K., Suda T. (2007). Function of oxidative stress in the regulation of hematopoietic stem cell-niche interaction. Biochem. Biophys. Res. Commun..

[57] Okita K., Yamanaka S. (2006). Intracellular signaling pathways regulating pluripotency of embryonic stem cells. Curr. Stem Cell Res. Ther..

[58] Staerk J., Constantinescu S.N. (2012). The JAK-STAT pathway and hematopoietic stem cells from the JAK2 V617F perspective. Jak-Stat.

[59] Fasouli E.S., Katsantoni E. (2021). JAK-STAT in early hematopoiesis and leukemia. Front. Cell Dev. Biol..

[60] Zeng J., Yi D., Sun W., Liu Y., Chang J., Zhu L., Zhang Y., Pan X., Dong Y., Zhou Y. (2021). Overexpression of HOXA9 upregulates NF-κB signaling to promote human hematopoiesis and alter the hematopoietic differentiation potentials. Cell Regen..

[61] Krstic A., Mojsilovic S., Jovcic G., Bugarski D. (2012). The potential of interleukin-17 to mediate hematopoietic response. Immunol. Res..

[62] Broxmeyer H.E. (2008). Chemokines in hematopoiesis. Curr. Opin. Hematol..

[63] Land S.C., Tee A.R. (2007). Hypoxia-inducible factor 1α is regulated by the mammalian target of rapamycin (mTOR) via an mTOR signaling motif. J. Biol. Chem..

[64] Cheng J.Q., Lindsley C.W., Cheng G.Z., Yang H., Nicosia S.V. (2005). The Akt/PKB pathway: Molecular target for cancer drug discovery. Oncogene.

[65] Carvalheira J.B., Ribeiro E.B., Folli F., Velloso L.A., Saad M.J. (2005). Interaction between leptin and insulin signaling pathways differentially affects JAK-STAT and PI 3-kinase-mediated signaling in rat liver. Biol. Chem..

[66] Santamaria K., Desmots F., Leonard S., Caron G., Haas M., Delaloy C., Chatonnet F., Rossille D., Pignarre A., Monvoisin C. (2021). Committed Human CD23-Negative Light-Zone Germinal Center B Cells Delineate Transcriptional Program Supporting Plasma Cell Differentiation. Front. Immunol..

[67] Aranda C.J., Gonzalez-Kozlova E., Saunders S.P., Fernandes-Braga W., Ota M., Narayanan S., He J.S., Del Duca E., Swaroop B., Gnjatic S. (2023). IgG Memory B Cells Expressing IL4R and FCER2 Are Associated with Atopic Diseases. Allergy.

[68] Chang L.S., Ming-Huey Guo M., Lo M.H., Kuo H.C. (2021). Identification of Increased Expression of Activating Fc Receptors and Novel Findings Regarding Distinct IgE and IgM Receptors in Kawasaki Disease. Pediatr. Res..

[69] Xu X., Chen S., Zhao Z., Xiao X., Huang S., Huo Z., Li Y., Tu S. (2021). Consolidative Hematopoietic Stem Cell Transplantation After CD19 CAR-T Cell Therapy for Acute Lymphoblastic Leukemia: A Systematic Review and Meta-analysis. Front. Oncol..

[70] Silva-Gomes R., Mapelli S.N., Boutet M.A., Mattiola I., Sironi M., Grizzi F., Colombo F., Supino D., Carnevale S., Pasqualini F. (2022). Differential Expression and Regulation of MS4A Family Members in Myeloid Cells in Physiological and Pathological Conditions. J. Leukocyte Biol..

[71] Liu F., Yuan Y., Bai L., Yuan L., Li L., Liu J., Chen Y., Lu Y., Cheng J., Zhang J. (2021). LRRc17 Controls BMSC Senescence Via Mitophagy and Inhibits the Therapeutic Effect of BMSCs on Ovariectomy-induced Bone Loss. Redox Biol..

[72] Li S., Yao J.C., Oetjen K.A., Krambs J.R., Xia J., Zhang J., Schmidt A.P., Helton N.M., Fulton R.S., Heath S.E. (2022). IL-1β Expression in Bone Marrow Dendritic Cells is Induced by TLR2 Agonists and Regulates HSC Function. Blood.

[73] Pietras E.M., Mirantes-Barbeito C., Fong S., Loeffler D., Kovtonyuk L.V., Zhang S., Lakshminarasimhan R., Chin C.P., Techner J.M., Will B. (2016). Chronic Interleukin-1 Exposure Drives Haematopoietic Stem Cells Towards Precocious Myeloid Differentiation at the Expense of Self-renewal. Nat. Cell Biol..

[74] Fok E.T., Moorlag S.J., Negishi Y., Groh L.A., Dos Santos J.C., Gräwe C., Monge V.V., Craenmehr D.D., van Roosmalen M., da Cunha Jolvino D.P. (2024). A Chromatin-regulated Biphasic Circuit Coordinates IL-1β-mediated Inflammation. Nat. Genet..

[75] Lin X., Lv X., Li B., Meng Q., Lai H., Gong Q., Tong Z. (2003). Heterogeneity of T cells in periapical lesions and in vitro validation of the proangiogenic effect of GZMA on HUVECs. Int. Endod. J..

[76] Lieberman J. (2020). Granzyme A activates another way to die. Immunol. Rev..

[77] Metkar S.S., Menaa C., Pardo J., Wang B., Wallich R., Freudenberg M., Kim S., Raja S.M., Shi L., Simon M.M. (2008). Human and mouse granzyme A induce a proinflammatory cytokine response. Immunity.

[78] Zhou Z., He H., Wang K., Shi X., Wang Y., Su Y., Wang Y., Li D., Liu W., Zhang Y. (2020). Granzyme A from cytotoxic lymphocytes cleaves GSDMB to trigger pyroptosis in target cells. Science.

[79] Seiler K., Minder P., Mashimo I., Humbert M., Simpson E., Tschan M.P., Torbett B.E. (2018). Hexokinase proteins impart distinct functions in myeloid development and cell death. Blood.

[80] Casirati G., Cosentino A., Mucci A., Salah Mahmoud M., Ugarte Zabala I., Zeng J., Ficarro S.B., Klatt D., Brendel C., Rambaldi A. (2023). Epitope editing enables targeted immunotherapy of acute myeloid leukaemia. Nature.

[81] Lei Y., Klionsky D.J. (2023). MCOLN3/TRPML3 bridges the regulation of autophagosome biogenesis by PtdIns3P and the calcium channel. Autophagy.

[82] Kim S.W., Kim D.H., Park K.S., Kim M.K., Park Y.M., Muallem S., So I., Kim H.J. (2019). Palmitoylation controls trafficking of the intracellular Ca^2+^ channel MCOLN3/TRPML3 to regulate autophagy. Autophagy.

[83] Ragland S.A., Criss A.K. (2017). From bacterial killing to immune modulation: Recent insights into the functions of lysozyme. PLoS Pathog..

[84] Qin P., Pang Y., Hou W., Fu R., Zhang Y., Wang X., Meng G., Liu Q., Zhu X., Hong N. (2021). Integrated decoding hematopoiesis and leukemogenesis using single-cell sequencing and its medical implication. Cell Discov..

[85] Gowhari Shabgah A., Al-Obaidi Z.M.J., Sulaiman Rahman H., Kamal Abdelbasset W., Suksatan W., Bokov D.O., Thangavelu L., Turki Jalil A., Jadidi-Niaragh F., Mohammadi H. (2022). Does CCL19 act as a double-edged sword in cancer development?. Clin. Exp. Immunol..

